# Current Status and Changes in Pain and Activities of Daily Living in Elderly Patients with Osteoarthritis Before and After Unilateral Total Knee Replacement Surgery

**DOI:** 10.3390/jcm8020221

**Published:** 2019-02-08

**Authors:** Yen-Feng Lai, Pei-Chao Lin, Chung-Hwan Chen, Jyu-Lin Chen, Hsin-Tien Hsu

**Affiliations:** 1 Department of Internal Medicine, Kaohsiung Medical University Hospital, Kaohsiung Medical University, No.100, Tz-You 1st Road, Kaohsiung 807, Taiwan; lai780452@gmail.com; 2 School of Nursing, College of Nursing, Kaohsiung Medical University, No.100, Shih-Chuan 1st Road, Kaohsiung 807, Taiwan; pclin@kmu.edu.tw; 3 Department of Medical Research, Kaohsiung Medical University Hospital, Kaohsiung Medical University, Kaohsiung 807, Taiwan; 4 Orthopedic Research Center, Kaohsiung Medical University, Kaohsiung 807, Taiwan; chchen@kmu.edu.tw; 5 Department of Orthopedics, Kaohsiung Medical University Hospital, Kaohsiung Medical University, Kaohsiung 807, Taiwan; 6 Departments of Orthopedics, College of Medicine, Kaohsiung Medical University, Kaohsiung 807, Taiwan; 7 Department of Orthopedics, Kaohsiung Municipal Ta-Tung Hospital, No.68, Jhonghua 3rd Rd, Cianjin District, Kaohsiung 801, Taiwan; 8 Department of Family Health Nursing, University of California San Francisco, 2 Koret Way San Francisco, CA 94143, USA; Jyu-Lin.Chen@ucsf.edu

**Keywords:** osteoarthritis, unilateral total knee replacement surgery, pain, activities of daily living

## Abstract

Knee osteoarthritis (OA) is a very common disease in the elderly, and total knee replacement (TKR) surgery is currently considered the most effective treatment. A prospective, observational, repeated measures study was performed to explore the current status and changes in pain and activities of daily living (ADL) in 58 OA elderly patients undergoing unilateral TKR. The Wong–Baker Faces Pain Rating Scale (WBS) for pain and the self-reported Barthel Index for ADL were measured on the day before surgery, 48 hours after surgery, and the day before discharge. Moderate pain was reported before surgery. Pain significantly improved after surgery and before discharge. At all three time points, pain scores were significantly higher in patients who used assistive devices compared to those who did not. Partial independence in ADL was reported before surgery. The ADL scores reported were highest before surgery, and those reported after surgery were lowest. However, ADL scores gradually increased before discharge. ADL scores were higher in the subjects who lived in a detached, single-family homes compared to those who lived in bungalows at all three time points. The results could be used to screen for knee OA elderly patients at high-risk for pain or low ADL and to provide timely intervention strategies as soon as possible.

## 1. Introduction

In the elderly, osteoarthritis (OA) is the most common form of arthritis in the knee and is prevalent in East Asia countries. The prevalence of knee OA is higher among women than men and increases with age [1,2]. The estimated prevalence rate of knee OA in people over 65 years old in the United States has reached as high as 12% [2] and is about 15% in individuals in Taiwan. In Taiwan, 3.5 million patients suffer from painful knee OA [3].

Total knee replacement (TKR) surgery is currently considered the most effective treatment for patients with knee OA [2]. In Taiwan, TKR surgery is covered by the national health insurance system and has been increasing substantially over the past decades [2]. Between 1996 and 2010, the rate of TKR tripled, and the incidence rates of TKR increased 2.7 times in men and 3.1 times in women [2]. Currently, the average hospital stay for TKR surgery is approximately 6 days. In knee OA patients, those who choose to undergo bilateral TKR tend to be young. Elderly knee OA patients often suffer from other chronic diseases. Therefore, unilateral TKR will be suggested to elderly knee OA patients after evaluation by the doctor [4]. Elderly OA patients are often willing to undergo surgery because their knee OA severely limits their mobility and/or activities of daily living (ADL). TKR, by replacing damaged knee joints with metal artificial knee joints and gaskets, can improve long-term joint pain, improve the range of movement, restore knee function and walking ability [5], and relieve pain, all of which improve performance of ADL and overall quality of life [6]. Many patients who undergo TKR surgery remain bedridden after surgery due to severe wound pain. Insufficient knee joint function can then limit ADL [7,8].

The main symptoms of knee OA include experiencing pain particularly when patients moving their knee or at the end of the day, stiffness especially after rest, local hard and soft swelling, and knee joint deformation [9]. Chronic pain caused by knee stiffness, weakness, swelling, and deformation is a typical clinical indicator of knee OA and can limit mobility. Increased physical activity can aggravate knee pain, whereas resting can ease it. As the severity of knee OA progresses, patients may experience chronic pain even without movement [10]. It also can cause the inability to walk, impairment of ADL, and reduced social interaction, all of which reduce quality of life and increase medical costs and use of healthcare resources [11]. Knee OA pain is chronic pain, whereas the pain experienced after TKR is acute pain caused by damage to muscles, bones, and tissues. An improvement in acute pain indicates that the injury is being repaired. The pain assessment tool recommended by the American Pain Society is the Numeric Rating Scale (NRS). The NRS is also recommended by the Gerontological Society of America (2002).

The relationships between demographic characteristics and pain have been discussed in literature. A previous study of factors related to severity of pain experienced by 1753 knee OA patients over a 6-year period found that risk factors for pain in these patients included obesity, chronic disease, female gender, non-white ethnicity, low education level, and young age [6]. In another study, Silverwood et al. systematically reviewed data for knee OA risk factors reported for OA patients older than 50 years in 46 studies. They concluded that the main causes of knee OA were overweight status (OR 1.98; 95% CI 1.57–2.20), obesity (OR 2.66; 95% CI 2.15–3.28), and female gender (OR 1.68; 95% CI 1.37–2.07) [12]. Several other longitudinal studies have focused on pain changes in knee OA patients before TKR and at varying time points after TKR [13]. For example, Lee et al., (2017) studied pain in 63 patients before and after TKR surgery, at 6 months and 12 months after they completed surgery. The mean pain score (on a scale from 0 to 10) significantly improved from a score of 7 before surgery to a score of 3 at 6 months after surgery, which was a significant improvement [13].

Although some ADL problems are expected shortly after TKR surgery, improvements in ADL are expected over the long term. The Barthel Index (BI) indicates the extent to which the subject can independently perform daily self-care tasks, e.g., eating, dressing, undressing, bathing, brushing hair, etc. [14,15]. Various methods of measuring ADL have been applied in clinical treatment and research [15]. For example, Liu (2004) performed a detailed and clear assessment of ADL by combining the Katz index and BI [15]. Another study by Tekin (2012) surveyed the expectations and self-reported improvements in ADL in 131 patients who had undergone TKR surgery [16]: 106 (80.9%) reported improved ADL, 114 (87%) reported improved pain, and 109 (83.2%) reported improved walking ability [16].

The relationships between demographic characteristics and ADL have also been discussed in literature. One study found that old age, overweight status, and poor ADL before TKR surgery can predict ADL after TKR surgery [12]. However, there are contradicting results regarding gender differences in ADL after TKR surgery. Chronic diseases often occur in older aged patients or males. More chronic diseases are prone to having postoperative complications and prolonged hospital stays, affecting TKR postoperative outcomes, so postoperative daily activities are poorer and increase the risk of death [17]. In another longitudinal study, 1260 elderly people (average age, 67 years) who had undergone TKR were tracked for 5.7 years. All patients were satisfied with their improvement in function after TKR. However, males had better ADL indices compared to females [18]. In postmenopausal knee OA women, joint degeneration, inflammation, and pain caused by hormonal changes can impair ADL [19].

Most recent studies of the effects of pain on ADL after TKR surgery have focused on analysis of long-term follow-up data for pain [7,20]. Elderly people who undergo TKR surgery often report acute pain after surgery, which limits their ability to perform ADL, e.g., turning over in bed and getting out of bed [7]. Therefore, potential limitation of ADL is another problem that should be carefully considered by patients receiving TKR. Despite the effects of these changes on surgery outcomes, including discharge and prognosis, few studies have compared pain and ADL before and after TKR surgery. Consequently, this study of elderly patients who required TKR surgery for treatment of knee OA had three objectives: (1) to collect data for pain and ADL status before surgery (Time 1), 48 h after surgery (Time 2), and before hospital discharge (Time 3); (2) to compare changes in pain and changes in ADL at the three time points; and (3) to compare pain and ADL in patients with different demographic and clinical characteristics at the three time points.

## 2. Materials and Methods

### 2.1. Design and Sample

A prospective, observational, repeated measures study design was used to investigate changes in pain and ADL during hospitalization after TKR surgery. A sample of consecutive knee OA patients was recruited from the orthopedics ward of a medical center in southern Taiwan. Data were collected with a structured questionnaire. The inclusion criteria were referral for unilateral TKR surgery, age 65 years or older, and ability to communicate in Mandarin or Taiwanese. Patients who met the DSM-IV criteria for mental illness were excluded. Other exclusion criteria were history of stroke, visual impairment, bilateral TKR surgery, cancer, other pain problems, rheumatoid arthritis, or physical disability.

The study purpose, process, and measurement procedure were explained to all participants who met the inclusion criteria, and written consent was obtained before data collection. The questionnaires were used to collect demographic data, ADL scale score, and pain scale score. Data collection was performed at three points: before surgery (Time 1, T1), 48 h after surgery (Time 2, T2), and before discharge (Time 3, T3) (after 6 days in hospital). The questionnaire required 15–20 min to complete.

The study was approved by the Institutional Review Board (KMU-IRB-20120289). Demographic and clinical data, the Barthel Index of Self-Reported ADL, and the Wong–Baker Faces Pain Rating Scale (WBS) were used in this study. In addition, one open question was added and helped to probe for more information regarding patients’ experience with pain or ADL change.

### 2.2. Instruments

The Index of Self-Reported ADL consists of 14 items. Each item is scored from 1 to 7 points, where 7 indicates complete independence (no assistance required) and 1 indicates complete dependence (subject can only perform <25% of the task or is unable to perform the task). Total scores range from 14 to 98 points. Higher scores indicate greater function in ADL: 14 to 20 points indicate complete dependence, 21 to 40 points indicate high dependence, 41 to 60 points indicate moderate dependence, 61 to 80 points indicate moderate independence, 81 to 97 points indicate high independence, and 98 points indicate complete independence. The original ADL scale for assessing functioning after TKR surgery had a Cronbach’s α of 0.86 [15].

The WBS is used to measure pain on a numerical scale (0, 2, 4, 6, 8, or 10). For each number on the scale, the instrument shows a picture of a face to provide a visual depiction of the pain level. The number 0 and a smiling face denote no pain, while the number 10 and a crying face denote the most severe pain. The participants were instructed to indicate the intensity of their pain by circling either the face or the number [21]. The WBS has demonstrated acceptable reliability and validity for clinical use [22]. 

### 2.3. Statistical Analysis

The sample size was calculated using G Power statistical software version 3.1 with an effect size of 0.3. For a two-tailed α of 0.05 and a power of 0.8, a sample size of 58 was adequate. Assuming an attrition rate of 10%, a sample size of at least 64 was required. The obtained data were coded and entered into a computer for analysis with SPSS (IBM Corp. Released 2011. IBM SPSS Statistics for Windows, Version 20.0. Armonk, NY: IBM Corp.). A *p* value <0.05 was considered statistically significant. To show the distribution of data, mean/median and standard deviation were used to represent continuous data. Numbers and percentage were used to describe categorical data. At T1, T2, and T3, repeated measures one-way analysis of variance was used to analyze changes in pain and ADL in the elderly knee OA patients, and repeated measures two-way analysis of variance was used to determine whether pain and ADL were associated with demographic or clinical characteristics. The Scheffe’s least significant difference (LSD) test for consequence comparison was performed for major factors that were statistically significant.

## 3. Results

A total of 64 eligible knee OA patients were recruited. Of these patients, three refused interviews due to severe postoperative pain, one refused due to postoperative acute renal failure requiring dialysis, and two refused due to fever caused by postoperative urinary tract infection. The resulting valid sample size of 58 represented a completion rate of 90.6%. Most participants (79.3%, *n* = 46) were female. The mean age of the 58 participants was 74.47 (SD = 4.96) years. The age range was 66 to 83 years (Table 1). Most were married (62.1%, *n* = 36), and most had an education level below elementary school (79.3%, *n* = 46).

### 3.1. Pain

Notably, preoperative pain significantly differed by education level (*p* = 0.02). The mean pain score for patients who were illiterate (7.09 ± 1.82) was significantly (*p* = 0.01) higher than that for patients who had a junior high school education or higher (5.25 ± 2.01). Table 2 shows that the WBS revealed significant differences in mean pain scores (F = 146.75, *p* < 0.001) at T1 (6.36 ± 1.88), at T2 (5.74 ± 1.93), and at T3 (3.28 ± 1.31). An LSD post-hoc analysis showed that the pain score at T3 was significantly lower than that at T1 (*p* < 0.001) and that at T2 (*p* < 0.001). Table 3 further shows that the pain scores at the three time points significantly differed by ethnicity (F = 4.97, *p* = 0.01). At all three time points, Minnan patients had higher pain scores compared to Mainland Chinese patients. The LSD post-hoc analysis also showed that Minnan patients had significantly higher pain scores compared to Mainland Chinese patients (*p* = 0.003). Hakka patients also had significantly higher pain scores compared to Mainland Chinese patients (*p* = 0.04). Additionally, the pain scores at the three time points significantly differed by use of assistive devices (*p* = 0.04) (Table 3). At all three time points, subjects who used assistive devices had higher pain scores compared to those who did not.

### 3.2. ADL

Preoperative ADL significantly differed by gender (*p* = 0.001). The total mean ADL score in women (92.98 ± 6.58) was significantly (*p* < 0.001) lower than that in men (96.75 ± 1.86). Additionally, the total mean ADL score in patients who did not use assistive devices was significantly higher than that in patients who used assistive devices (*p*< 0.001). Table 2 shows that mean ADL scores were 93.76 (± 6.10) at T1, 73.64 (± 5.69) at T2, and 77.52 (± 9.45) at T3. Before surgery, the ADLs with the three lowest mean scores were—in descending order—climbing stairs, walking inside a room, going to/getting on and off a toilet, and moving from bed to chair and back again. At T2 and T3, the ADLs with the three lowest mean scores were—in descending order—climbing stairs, bathing, and moving from bed to chair and back. The ADL scores significantly differed among the three time points (F = 123.09, *p* < 0.001). In LSD post-hoc analysis, ADL scores at T1 were significantly higher than those at T2 and T3 (*p* < 0.001; *p* < 0.001). The ADL scores at T3 were significantly higher than those at T2 (*p* < 0.001) (Table 2).

Table 4 shows that the ADL scores at the three time points significantly differed by age (F = 3.62, *p* = 0.02). Table 4 shows that, in LSD post-hoc analysis, ADL scores in the 65–69 years age group (*p* = 0.007) and the 75–79 years age group were significantly (*p* = 0.004) higher than the ADL scores in the 80+ years age group. Table 4 also shows that the ADL scores at the three time points significantly differed by living environment (*p* = 0.02). Specifically, those who lived in single-family homes had higher ADL scores than those who lived in bungalows (Table 4). Finally, Table 4 shows that the ADL scores significantly differed by use of assistive devices, and significant interacting effects between ADL and use of assistive devices were observed (F = 4.21, *p* = 0.04). At T1 and T3, patients who did not use assistive devices had higher ADL scores than those who did use assistive devices. At T3, however, patients who used assistive devices had higher ADL scores than those who did not use assistive devices.

## 4. Discussion

The demographic characteristics of the study were similar to those in previous studies [16,23]. Chen (2011) investigated perceptions and coping strategies in 104 elderly patients treated for knee OA at a medical center in Taiwan. Pain scores were highest in those who were illiterate, possibly due to their lack of knowledge and limited access to medical information [24]. If the pain in a knee joint affects ADL and self-care before surgery, the patient is likely to report moderate to severe pain levels. In the current study, the mean score for preoperative pain was 6.36 ± 1.88, which indicated a moderate level of pain. The main impact of pain was fear of turning over in bed and getting out of bed, which is consistent with previous reports [24].

In most of the previous knee OA studies, the duration of follow up was long, and the reported improvement tended to be most obvious at 6 months after surgery [13]. Although the current study had a relatively short follow up, significant reductions in pain were observed, as reported previously in Wu, Tang, and Hong (2016). In that study, the authors used a longitudinal design to investigate 75 knee OA patients who had received surgery for artificial knee joints in a central medical center [10]. The severity of pain was measured by visual analog scale at 24, 48, and 72 h postoperatively. Postoperative pain was generally moderate (66%) and significantly decreased over time (*p* < 0.001). In the current study, the pain level measured at the three time points significantly differed by ethnicity (*p* = 0.01). At all three time points, pain levels were higher in Minnan patients compared to Mainland Chinese patients. The likely reason is that most of the participants in the current study were Minnan. Future research should investigate pain in more ethnic groups. Additionally, the pain levels at the three time points significantly differed between subjects who did and did not use assistive devices. At all three time points, patients who used assistive devices had significantly higher pain levels compared to those who did not use assistive devices. Subjects who use assistive devices tend to have high pain scores because they usually have pain caused by poor joint function.

The mean total scores for preoperative ADL indicated partial independence in this study. At T1, the ADL items that had the three lowest scores were, in descending order, climbing two to three steps of stairs, walking inside a room, using/getting on and off a toilet, and moving from bed to chair and back. For elderly knee OA patients with knee weakness and pain, the most difficult task was climbing stairs because they could only rely on handrails for added support. At T2 and T3, the ADL items with the three lowest mean scores were—in descending order—climbing stairs, bathing, and moving from bed to chair and back again. Patients who have received TKR surgery have difficulty performing tasks that require stretching in the knee. Climbing stairs was again the most difficult task to perform. Additionally, while undressing and bathing, they may have had difficulty keeping the wound dry and cannot easily bend their knees due to swelling. When moving from bed to chair, they required assistance in moving the affected limb. The use of assistive devices for walking after surgery progressively improved functioning. Kim et al. (2010) tracked 372 Korean women for 12 months after they had received TKR [25]. Of these, 261 (70.2%) of the women completed follow up. The authors found that, although the subjects had difficulty performing knee flexion after surgery, they significantly improved in ADL, such as climbing stairs, sitting in a chair, and standing up from a chair [25].

In the current study, the level of ADL at the three time points significantly differed by age. The ADL scores were lowest in patients aged 80 years or older, which is consistent with the literature [16,26]. The results of this study suggest that, regardless of age category, a mean ADL score of 74 or higher is a reasonable and objective criterion for discharge. Older patients are likely to have severe degenerative knee problems [9]. Additionally, those who lived in detached, single-family homes had higher ADL scores compared to those who lived in bungalows at all three time points. Most participants in the current study were residents from southern Taiwan, who usually live in a detached, single-family homes. Their rooms were usually on the second floor, so they had to climb stairs every day. They decided to receive surgery after experiencing pain. After surgery, the temporary swelling in the knees gradually improved, and stair climbing exercises were gradually added to their exercise routines. The patients were accustomed to a specific living environment and were unlikely to relocate. Therefore, they were motivated to participate in the rehabilitation program after surgery so that they could quickly adapt to their living environment before discharge. Therefore, those who lived in detached, single-family homes were likely to have high ADL scores [27]. 

The ADL scores significantly differed by use of assistive devices, and ADL had significant interacting effects with the use of assistive devices. At T1 and T2, ADL scores were lower in patients who had used assistive devices compared to those who had not. Those who had used assistive devices preoperatively due to lack of lower extremity muscle strength or unstable gait were likely to use assistive devices after surgery [28]. At T3, however, those who had used assistive devices obtained higher ADL scores than those who had not. The likely reason is that those who required assistive devices before surgery had poor knee function and poor ADL function. After TKR surgery, however, those who used assistive devices to support their body weight substantially reduced stress in the wound area, which improved knee function. Patients who had experience using assistive devices were likely to adapt to postoperative conditions quickly, which resulted in better ADL before discharge [24,28].

In contrast with previous studies, the ADL scores at the three time points did not significantly differ by gender or by BMI. In previous studies, women have had lower ADL compared to men both before and after surgery, and ADL has shown significant correlations with gender [19]. Compared to subjects in studies performed in other countries, the subjects in the current study had less obesity and smaller variances in BMI. A BMI higher than 30 kg/m^2^ is reportedly associated with low ADL levels after TKR surgery [12,29]. Another study compared the impact of obesity (30 ≤ BMI < 40 kg/m^2^) and morbid obesity (40 ≤ BMI < 50 kg/m^2^) on ADL after TKR surgery. The authors reported that ADL level was significantly lower in those BMI more than 40 kg/m^2^ [2,30].

A noted limitation of this study was the use of a consecutive sample of patients treated at a single medical center in south Taiwan, which might have contributed to biased the results. In future studies, stratified random sampling is recommended to obtain a sample that represents the overall population. Additionally, the subjects in this study had a high average age. Therefore, some required the assistance of family members to complete the questionnaire. For some participants, the researcher needed to review medical records to obtain correct medical information.

## 5. Conclusions

This study of knee OA patients undergoing TKR surgery found that those with lower levels of education had a lower pain threshold compared to those with higher levels of education. Therefore, the use of simple language and pictures are suggested to when communicating with TKR surgery patients about pain assessment and pain management [24]. Additionally, patients who had used assistive devices were much more effective in self-care after surgery. Therefore, elderly patients should be advised to use assistive devices to reduce stress on the wound and promote recovery of the knee joint. Additionally, due to wound swelling and adaptation to the artificial knee point, TKR patients may require assistance (e.g., assistance in moving the affected limb or bending of the knee). 

Even participants who had only received a unilateral TKR had difficulty regaining mobility. To improve these problems, these patients should be advised to use handrails when walking up and down stairs or to avoid using stairs. These patients would also benefit from lower limb muscle training exercises to increase their lower limb muscle strength. After surgery, the patients should wear loose clothing and be careful to keep the wound dry and clean. During bathing, they should use a chair or other device to support their body weight. Long-handled sponges can also make bathing easier. In summary, assistive devices are very useful for patients undergoing rehabilitation. Such devices can accelerate their recovery to normal ADL and can reduce the risk of falls. 

According to the results of this study, performance of ADL was worst after surgery and improved before hospital discharge. Although not all patients can be expected to achieve the same mobility, knee function, and ADL levels they had before surgery, improvement in ADL is a useful indicator when TKR patients are evaluated for discharge. In future studies, data should be collected at more time points before surgery, including 48 h after TKR surgery, before discharge, and then 1 month, 3 months, 6 months, and 1 year after surgery. The additional data collection would provide a trajectory of data that could be used to evaluate the benefit of TKR surgery and improve recovery. Medical professionals can use the results of this study to screen for high-risk individuals, provide timely intervention strategies, and assist patients in returning to normal ADL as soon as possible.

## Figures and Tables

**Table 1:** Demographic data.

Variables	Subgroups	Number of Subjects (N)	Percentage (%)	Mean/median ± SD
Gender	Male	12	20.7	
	Female	46	79.3	
Age, years	65–69	13	22.4	74.47 ± 4.96
	70–74	16	27.6	
	75–79	21	36.2	
	>80	8	13.8	
Marital status	Married	36	62.1	
	Single	22	37.9	
Education level	Illiterate	22	37.9	
	Junior high school	24	41.4	
	Higher education	12	20.7	
Body mass index	18.5–24	10	27	26.30 ± 3.98
	24–26.9	23	17.2	
	>27	25	39.7	
Religion	nil	9	15.5	
	Buddhist	46	79.2	
	Christian	3	5.2	
Race	Minnan	47	81	
	Mainland Chinese	3	5.2	
	Hakka	8	13.8	
Living status	Living with family	56	96.9	
	Living alone	2	3.4	
Use of assistive devices	No	39	67.2	
	Yes	19	32.8	

**Table 2:** Status and changes in pain and activities of daily living (ADL) at three time points (*n* = 58).

Variable	T1 Mean ± SD	T2 Mean ± SD	T3 Mean ± SD		F Value	Difference Between the Two Groups
Pain	6.36 ± 1.88	5.74 ± 1.93	3.28 ± 1.31		146.75 ***	1 > 3 (*p* < 0.001)
						2 > 3 (*p* < 0.001)
ADL	93.76 ± 6.10	73.64 ± 5.69	77.52 ± 9.45		123.09 ***	1 > 2 (*p* < 0.001)
						1 > 3 (*p* < 0.001)
						3 > 2 (*p* < 0.001)

T1: Before surgery, T2: 48 h after surgery; T3: before discharge; 1: before surgery, 2: 48 h after surgery, and 3: before discharge; *** *p* < 0.001.

**Table 3:** Pain scores compared by demographic characteristics in patients who had received unilateral total knee replacement surgery at three points in time (*n* = 58). LSD = least significant difference.

Variables	T1 Mean ± SD	T2 Mean ± SD	T3 Mean ± SD	F Value	*p* Value	LSD Post-hoc Analysis
Race				4.97	0.01	1 > 2 (*p* = 0.003)
1. Minnan	6.57 ± 1.84	5.85 ± 1.81	3.49 ± 1.16			3 > 2 (*p* = 0.004)
2. Mainland Chinese	4 ± 0	4 ± 1.73	1.33 ± 1.53			
3. Hakka	6 ± 1.93	5.55 ± 2.55	2.75 ± 1.49			
Aids use				4.65	0.04	
1. No	6.08 ± 1.75	5.51 ± 1.97	3.05 ± 1.05			
2. Yes	6.95 ± 2.04	6.21 ± 1.78	3.74 ± 1.66			

Race 1: Minnan, 2: Mainland Chinese, and **3**: Hakka.

**Table 4:** ADL scores compared by demographic characteristics in patients who had received unilateral total knee replacement surgery at the three time points (*n* = 58).

Variables	T1 Mean ± SD	T2 Mean ± SD	T3 Mean ± SD	F value	*p* value	LSD Post-hoc Analysis
Age				3.62	0.02	1 > 4 (*p* = 0.007) 3 > 4 (*p* = 0.004)
1. 65–69	95.85 ± 4.16	75.08 ± 2.99	78.69 ± 3.84			
2. 70–74	93.71 ± 6.50	73.58 ± 5.23	75.64 ± 16.37			
3. 75–79	94.57 ± 4.79	75.00 ± 3.18	79.62 ± 3.31			
4. ≧80	89.40 ± 8.47	69.00 ± 10.02	74.20 ± 10.23			
Living environment				5.49	0.02	
Bungalow	92.62 ± 7.98	71.15 ± 9.69	72.69 ± 17.64			
Single-family home	94.09 ± 5.51	74.36 ± 3.74	78.91 ± 4.69			
Use of assistive devices				4.21	0.04	
No	96.54 ± 1.89	74.21 ± 3.83	76.97 ± 10.30			
Yes	88.05 ± 0.70	72.47 ± 8.34	78.63 ± 0.54			
